# Impact of urban contamination of the La Paz River basin on thermotolerant coliform density and occurrence of multiple antibiotic resistant enteric pathogens in river water, irrigated soil and fresh vegetables

**DOI:** 10.1186/s40064-016-2132-6

**Published:** 2016-04-22

**Authors:** Violeta Poma, Nataniel Mamani, Volga Iñiguez

**Affiliations:** Instituto de Biología Molecular y Biotecnología, Universidad Mayor de San Andrés, Facultad de Ciencias Puras y Naturales, Campus Universitario-Cota Cota, La Paz, Bolivia

**Keywords:** Thermotolerant coliforms, Enteropathogenic bacteria, Antibiotic multi-resistance, Sewage water, River contamination, Contamination of soil and vegetables, Food and waterborne diseases risk, Amazon macrobasin

## Abstract

La Paz River in Andean highlands is heavily polluted with urban run-off and further contaminates agricultural lowlands and downstream waters at the Amazon watershed. Agricultural produce at this region is the main source of vegetables for the major Andean cities of La Paz and El Alto. We conducted a 1 year study, to evaluate microbial quality parameters and occurrence of multiple enteropathogenic bacteria (*Enterohemorrhagic E. coli*—EHEC, *Enteroinvasive E. coli* or *Shigella*—EIEC/*Shigella*, *Enteroaggregative E. coli*—EAEC, *Enteropathogenic E. coli*—EPEC *Enterotoxigenic E. coli*—ETEC and *Salmonella*) and its resistance to 11 antibiotics. Four sampling locations were selected: a fresh mountain water reservoir (un-impacted, site 1) and downstream sites receiving wastewater discharges (impacted, sites 2–4). River water (sites 1–4, N = 48), and soil and vegetable samples (site 3, N = 24) were collected during dry (April–September) and rainy seasons (October–March). Throughout the study, thermotolerant coliform density values at impacted sites greatly exceeded the guidelines for recreational and agricultural water uses. Seasonal differences were found for thermotolerant coliform density during dry season in water samples nearby a populated and hospital compound area. In contrast to the un-impacted site, where none of the tested enteropathogens were found, 100 % of surface water, 83 % of soil and 67 % of vegetable samples at impacted sites, were contaminated with at least one enteropathogen, being ETEC and *Salmonella* the most frequently found. ETEC isolates displayed different patterns of toxin genes among sites. The occurrence of enteropathogens was associated with the thermotolerant coliform density. At impacted sites, multiple enteropathogens were frequently found during rainy season. Among isolated enteropathogens, 50 % were resistant to at least two antibiotics, with resistance to ampicillin, nalidixic acid, trimethoprim–sulfamethoxazole and tetracycline commonly present. Moreover, some *Salmonella* isolates were distinguished by their multi-resistance to ≥8 antibiotics, within soil and vegetable samples. Overall, this study demonstrates that La Paz River—an affluent of the Amazon macrobasin—is heavily polluted along the year with a high density of thermotolerant coliforms and is a reservoir of multiple antibiotic resistant enteropathogens, present in river water, soil and vegetables. These data highlight health risk associated with food and waterborne diseases at the region.

## Background

Worldwide, the contamination of fresh water resources due to urbanization affects food security and ecosystem sustainability (WWAP 2012). This situation is exacerbated in developing countries and more so in dry regions such as the Bolivian Highlands, where population growth, urban expansion and widespread malnutrition give rise to an increasing demand for water that is crucial for food security (Buxton et al. 2013). In this region, along with a shortage of water, urban wastewater directly drains into fresh water bodies that flow downstream into the Amazon macrobasin, contributing to its aquatic ecosystem degradation. Moreover, within agricultural dry regions, vegetable production is enhanced by the use of river surface water and sludge as sources of irrigation and organic nutrients (Duran et al. 2003). This is the case of the agricultural production along La Paz River basin, where river effluents are highly valued by farmers, allowing for a continuous crop production throughout the year, to respond to the growing food demand of the nearby cities of La Paz and El Alto, which are the biggest urban centers in the Bolivian Andean highlands (MMAyA 2012).

Water pollution is largely associated with the incidence of water and food borne diseases and the origin of many outbreaks (Buchholz et al. 2011; Juliana et al. 2008; Little and Gillespie 2008; Marcheggiani et al. 2015). Moreover, water–borne diseases are among the most common causes of mortality and morbidity around the world, accounting for approximately 3 % of all deaths, affecting primarily children in developing countries. It is estimated that each year around 2 million people die from diarrheal diseases, likely associated to contaminated food and water (WHO 2014). This situation may likely be exacerbated by climate change in the coming years, increasing human exposure to environmental pathogens (Boxall et al. 2009).

In Bolivia it is estimated that around 25 % of children less than five years of age suffer from acute diarrhea, and more than 870 thousand diarrheal cases are registered in all ages annually (SNIS 2013). It is unknown however, how many of these cases have a water and/or food borne origin.

In Bolivia, cholera epidemics started in 1992 at the agricultural area of La Paz River basin and extended to most of the country, reaching 44,000 reported cases. The cholerae outbreak was associated with the consumption of fresh vegetables irrigated with sewage polluted water (CDC 1993; MS 2000). Later in 1997, Ohno et al. reported the occurrence of fecal pollution and enteric pathogens in the study area (Ohno et al. 1997). Although no further large outbreaks were reported (INE 2011; SNIS 2013), consumption of raw vegetables cultivated in the area may be associated with a risk of diarrhea.

Pollution of La Paz River contributes to the environmental degradation of the Amazon watershed and may increase the health risk of downstream agricultural rural settlements and riverine communities, which are affected by water borne diseases (Confalonieri and Fonseca 2013; Rede Interagencial de Informações Para a Saúde 2012). Fecal pollution indicators associated with urban contamination and occurrence of enteric pathogens have already been described in the Amazon watershed (De Paula et al. 2007; Diniz-Mendes et al. 2008; Miagostovich et al. 2008; Pereira et al. 2010).

The aim of this study was to evaluate the microbial river water quality at un-impacted and impacted sites, as well as the occurrence of multiple enteropathogenic bacteria (*Enterohemorrhagic E. coli*—EHEC, *Enteroinvasive E. coli* or *Shigella*—EIEC/*Shigella* pathovar, *Enteroaggregative E. coli*—EAEC, *Enteropathogenic E. coli*—EPEC *Enterotoxigenic E. coli* and *Salmonella*) and its resistance to antibiotics in river water, soil and vegetable samples of the La Paz River basin, throughout a 1-year period.

## Methods

### Study area

La Paz River basin network is located in the northeast region of the high plateau in La Paz, Bolivia, extending from mountain glaciers towards urban and agricultural areas with altitudes ranging from 2400 to 5500 meters above sea level. La Paz River is part of the Amazon macrobasin, being one of the main Andean tributaries of the Madeira River (UNEP 2004; Goulding et al. 2003). Along the river’s course in La Paz city, urban and industrial wastewater discharges are directly released into surface water without prior treatment. Furthermore, at lowland agricultural areas, river water and sediments are used to irrigate and to flood vegetable crops.

### Sampling sites and sample collection

**Fig. 1:**
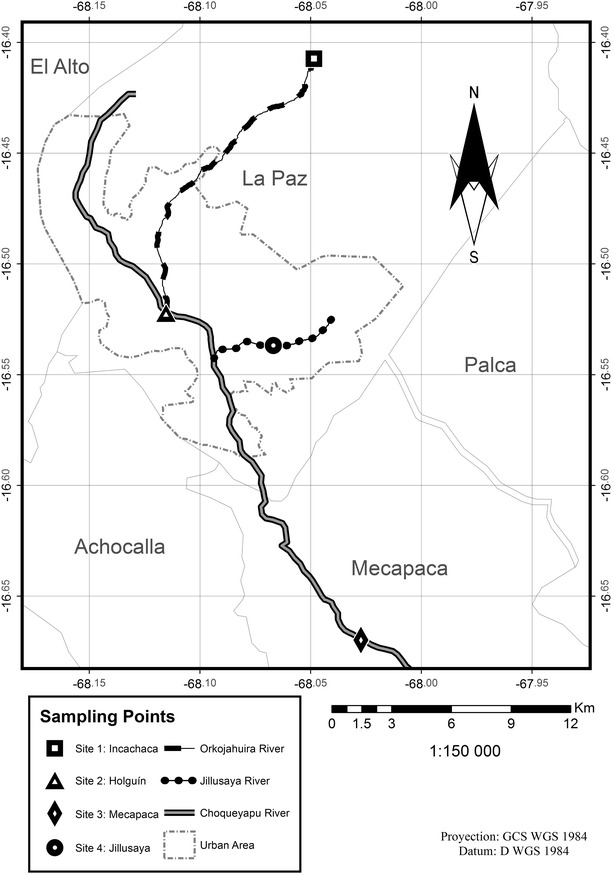
Sampling study area. *Site 1* Incachaca; *site 2* Holguin; *site 3* Mecapaca and *site 4* Jillusaya

Four sampling sites along the La Paz River basin were selected based on water quality, predominant source of pollution and use. Site 1, Incachaca, is located at the upstream un-impacted region, right at the exit point of the fresh water reservoir. Site 2, Holguín, is located 17.4 km downstream from site 1, at the impacted and heavily populated urban area right next to the hospital compound. Site 3, Mecapaca, is located at the agricultural lowland impacted region, placed 40 km from site 1. Site 4, Jillusaya River, is an impacted urban side affluent of the La Paz River (Fig. 1). Soil and vegetable samples were only collected at Mecapaca river shorelines (site 3).

Surface water, vegetable and soil samples were collected monthly between April 2013 and March 2014 at each sampling point, comprising dry (April–September) and rainy (October–March) seasons were collected at each site along the year (1 sample/month). The pH, temperature (°C), conductivity (μS/cm) and redox potential (mV) were measured in situ (Oakton Instruments, Vernon Hills) as described by EPA guidelines with three replicates (EPA 2007). Water samples were taken in sterilized 600 ml plastic bottles, following APHA and EPA guidelines (APHA 2005; EPA 2007). Green leafy vegetables (lettuce or chard) and soil samples were collected directly into sterilized plastic bags. Samples were kept at 4–6 °C upon transportation to the laboratory of the Instituto de Biología Molecular y Biotecnología (IBMB) and processed on the same day.

### Microbiological analysis

#### Thermotolerant coliform

Thermotolerant coliform is defined as the group of coliform bacteria which produces gas from lactose in 48 h at 44.5 °C (Resolution MEPC 2006). The bacterial density of thermotolerant coliform in water, vegetable and soil samples was evaluated by the multiple tube fermentation technique, using A1-media (Sigma-Aldrich, St. Louis) as described by APHA guidelines (APHA 2005) and reported as MPN/100 ml or MPN/g (in soil and vegetable samples).

### Enteropathogenic bacteria detection

Water samples (200 ml) and vegetable elution samples (30 g of vegetables washed in 200 ml of peptone water), were separately filtered using a 0.45 μm pore sized nitrocellulose membrane filter (Millipore-Sigma-Aldrich, St. Louis). Soil samples (3 g) and water and vegetable filters were inoculated into EC Broth at 37 °C, for 18 h.

#### ***Diarrheogenic Escherichia coli*** (DEC)

Overnight bacterial enrichment was streaked on to MacConkey agar plates (Difco Laboratories, Detroit) and incubated for 18 h at 37 °C. From each plate, five lactose positive and five lactose negative colonies were isolated in MacConkey agar and resuspended in tridistilled water and subjected to boiling for DNA extraction. Conventional PCR using specific primers for virulence markers was used to detect among lactose positive bacteria: stx1/stx2 (*Enterohemorrhagic E. coli*—EHEC), ipaH (*Enteroinvasive E. coli*/*Shigella* pathovar—EIEC/*Shigella*), paa (*Enteroaggregative E. coli*—EAEC), eae/bfp (*Enteropathogenic E. coli*—EPEC) and lt/sth/stp (*Enterotoxigenic E. coli*—ETEC) (Gonzales et al. 2013a; Moon et al. 2005; Rodas et al. 2009). In addition, lactose negative isolates were also tested to detect ipaH (*Enteroinvasive E. coli*/*Shigella* pathovar—EIEC/*Shigella*).

Reference strains that were used as positive controls included: 3b for (stx1/stx2), ATCC-43893 for (ipaH), O42 for (paa), 12b for (eae, bfp) and H17047 for (lt/sth/stp). All positive samples were confirmed twice by independent PCR assays. Negative controls (commensal *E. coli* ATCC 25922 and tri-distilled water) were included in PCR runs.

#### *Salmonella*

Overnight bacterial enrichment was inoculated into semisolid medium Rappaport–Vassiliadis (HiMedia Laboratories, Pennsylvania), and incubated for 48 h at 43 °C. Samples in which a whitish growing halo was observed were used for PCR analysis using primers for virulence marker invA (Rahn et al. 1992). Reference strains *Salmonella enterica* Serotype *Typhimurium* DT104 was used as positive control. Negative controls (tri-distilled water) were included in all PCR runs.

### Multiple enteropathogen index

The multiple enteropathogenic bacteria (MEB) index was calculated as follows: *a*/*b* × *c*, where *a* is the aggregate number of positive samples for any enteropathogenic bacteria at each sampling point, *b* is the number of pathogens evaluated in the study and *c* is the total number of samples.

### Antimicrobial susceptibility testing

Antibiotic susceptibility testing of pathogenic isolates was performed by the Kirby–Bauer disk diffusion method as described by the Clinical and Laboratory Standards Institute guidelines (CLSI 2007), using Mueller–Hinton agar (Difco Laboratories). The antibiotics disks (Oxoid, Hampshire) and their concentration were: ampicillin (AM 10 μg), ampicillin–sulbactam (AB 10 μg/10 μg), cefoxitin (FX 30 μg), cefotaxime (CT 30 μg), ciprofloxacin (CI 5 μg), chloramphenicol (C 30 μg), gentamicin (CN 10 μg), nalidixic acid (NA 30 μg), streptomycin (S 10 μg), tetracycline (TC 30 μg) and trimethoprim–sulfamethoxazole or cotrimoxazole (ST 1.25 μg/23.75 μg). *Escherichia coli* ATCC 25922 was used as the control strain.

The multiple antibiotic resistance (MAR) index was calculated for each isolate by this formula: *a/b*, where *a* is the number of antibiotics to which the isolate is resistant and *b* is the number of antibiotics evaluated in the study.

### Data analysis

All statistical analyses were performed using R software version 2.15.1 at *P* < 0.05 significance level. Significant differences in water quality parameters, between dry and rainy seasons were tested by Wilcoxon test. Principal Component Analyses (PCA) was used as an exploratory analysis to visualize differences of water parameters between the impacted and the non-impacted sites. Comparison of MEB index between seasons at impacted sites was achieved by Wilcoxon test.

## Results

### Microbiological and physicochemical parameters

**Fig. 2:**
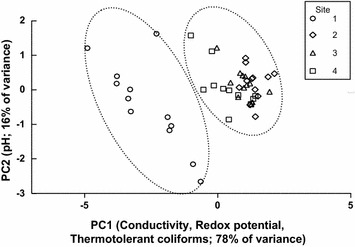
Principal component analysis (PCA) ordination plot based on physicochemical and microbiological parameters data from river water sampling sites, at La Paz River basin. The percentage of variation explained by *each axis* is shown

Microbiological and physicochemical parameters (thermotolerant coliforms, pH, temperature, conductivity and redox potential) markedly differed between the un-impacted and impacted sites (Table 1). As shown by Principal Component Analysis based on physicochemical and microbiological parameters, impacted sites 2, 3 and 4 cluster together away from un-impacted site 1. The first two components explained 98.06 % of variance; component 1 included thermotolerant coliforms, conductivity and redox potential, while component 2 pH (Fig. 2).

**Table 1:** Mean value and standard deviation for physicochemical and microbiological parameters, along La Paz River basin water, soil and vegetable sampling points

Site no./samples (N)	pH		Temperature (°C)		Conductivity (μS/cm)		Redox potential (mV)		Thermotolerant coliform density MPN^a,b^	
	Mean	SD	Mean	SD	Mean	SD	Mean	SD	Mean	SD
Water (N = 12)										
1	6.84	1.10	9.46	1.64	172.83	97.28	−25.15	38.87	3.73 × 10^1^	9.88 × 10^1^
2	7.93	0.43	12.73	1.87	1303.50	351.07	−82.62	6.62	*1.42* × *10* ^*6*^	1.90 × 10^6^
3	7.81	0.48	18.78	2.50	1216.90	341.74	−76.71	11.23	*3.08* × *10* ^*5*^	1.87 × 10^5^
4	7.64	0.65	ND	ND	750.48	253.04	−68.45	16.81	*3.05* × *10* ^*5*^	3.77 × 10^5^
3/soil (N = 12)	NA	NA	NA	NA	NA	NA	NA	NA	4.61 × 10^2^	7.51 × 10^2^
3/vegetable (N = 12)	NA	NA	NA	NA	NA	NA	NA	NA	6.85 × 10^1^	1.46 × 10^2^

MPN values shown in italics are above the standard safe water values, for use in recreational and agricultural activities

*NA* not applicable

*ND* not determined

^a^MPN (most probable number of thermotolerant coliforms)/100 ml in water samples

^b^MPN (most probable number of thermotolerant coliforms)/g in vegetable and soil samples

In water samples at all impacted sites, thermotolerant coliforms were continuously detected throughout the sampling year at much higher densities (10^5^–10^6^ MPN/100 ml) than the values found at site 1 (10^1^ MPN/100 ml) (Table 1). The range between the lowest (un-impacted site 1) and the highest (impacted site 2) thermotolerant coliform density, measured during the monitoring year reached 5 orders of magnitude. At impacted sites, the mean density of thermotolerant coliforms at the most contaminated site (site 2) was 1.42 × 10^6^ MPN/100 ml and at the less contaminated site (site 4) 3.05 × 10^5^ MPN/100 ml. Moreover, at all impacted sites, thermotolerant coliforms exceeded by at least one order of magnitude, the standards for either recreational or irrigation water.

Soil and vegetable samples irrigated with river water also presented thermotolerant coliform density ranging from 4.61 × 10^2^ MPN/g (soil) and 6.85 × 10^1^ MPN/g (vegetables) (Table 1).

Regarding seasonality, thermotolerant coliform density significantly differed between rainy and dry seasons at site 2, with higher densities at the latter (*P* < 0.05) (Table 2). At site 1, differences of mean pH and temperature values were observed between dry and rainy season (Table 2), while no seasonal differences among physicochemical data were found at impacted sites.

**Table 2:** Seasonal variation of physicochemical and microbiological parameters, along La Paz River basin water sampling points

Site no	Season	pH	Temperature (°C)	Conductivity (μS/cm)	Redox potential (mV)	MPN/100 ml^a^
1	Dry	*5.95*	*8.43*	163.23	−43.70	10.23
	Rainy	*7.72*	*10.49*	182.43	−45.92	11.77
2	Dry	7.67	12.13	1407.94	−86.15	*2.40* × *10* ^*6*^
	Rainy	8.19	13.32	1199.06	−79.09	*4.35* × *10* ^*5*^
3	Dry	7.60	17.99	1290.44	−81.13	3.55 × 10^5^
	Rainy	8.01	19.56	1143.39	−72.28	2.62 × 10^5^
4	Dry	7.38	10.95	713.47	−69.98	4.81 × 10^5^
	Rainy	7.90	17.49	781.33	−66.93	1.29 × 10^5^

Significant differences in water quality parameters between dry and rainy seasons (*P* < 0.05), calculated by Wilcoxon, are shown in italics

^a^Most probable number of thermotolerant coliforms

### Enteropathogenic bacteria distribution

Occurrence of enteropathogens clearly differed between un-impacted and impacted sites, since none of the tested pathogens were detected at the former (Table 3). On average 100, 83 and 67 % of polluted water, soil and vegetable samples respectively, harbored any of the tested enteropathogens.

**Table 3:** Percentage of enteropathogenic bacteria found at La Paz River basin, water, soil and vegetable sampling points

Site/sample	ETEC% (N)^a^	EPEC%^d^ (N)^a^	EAEC% (N)^a^	*Salmonella*% (N)^a^	EIEC/*Shigella*%^e^ (N)a	MEB^b^	Any pathogen%^c^
1/water	0	0	0	0	0	0	0
2/water	100 (12)	58 (7)	67 (8)	92 (11)	8 (1)	0.54	100
3/water	100 (12)	50 (6)	67 (8)	83 (10)	0	0.50	
4/water	83 (10)	33 (4)	50 (6)	92 (11)	17 (2)	0.46	
3/soil	67 (8)	25 (3)	33 (4)	33 (4)	33 (4)	0.32	83
3/vegetable	67 (8)	0	17 (2)	33 (4)	0	0.19	67

EHEC was not detected in any of the samples

^a^(N) is the number of positive samples detected for each pathogen (i.e. ETEC, EPEC, EAEC, *Salmonella* and EIEC/*Shigella*) at each site along the year. A total of 12 samples were collected at each site along the year (1 sample/month)

^b^Multiple enteropathogenic bacteria index (MEB)

^c^Percentage of positive samples for at least one enteropathogen detected

^d^All EPEC isolates were atypical (bfp negative)

^e^All EIEC/*Shigella* isolates were non-lactose fermenting colonies

Among enteropathogens, ETEC and *Salmonella* represented the most frequently found isolates followed by EAEC, EPEC and the EIEC/*Shigella* pathovar, whereas EHEC was not detected in any of the samples (Table 3). As shown in Table 5, ETEC isolated colonies displayed different toxin genes profiles along sites with a higher prevalence of LT/STh (63 %) toxins, while STp was found only in vegetable samples (25 %). All EPEC isolates were atypical (bfp negative) and all colonies assigned to EIEC/*Shigella* pathovar were non-lactose fermenting.

**Fig. 3:**
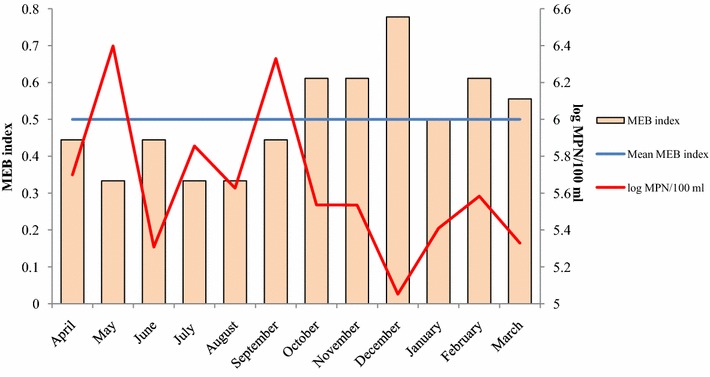
Monthly distribution of MEB index and thermotolerant coliforms, at impacted water samples of La Paz River basin along the study year. Mean MEB index was calculated for each month across sites (sites 2–4). Mean values of MEB index between dry and rainy season, displayed significant differences (Wilcoxon test, *P* < 0.05)

The monthly distribution of the multiple enteropathogenic bacteria (MEB) index at the impacted water sampling sites along the entire year is presented in Fig. 3. During the monitoring, the co-occurrence of different enteropathogenic bacteria was most frequent at the rainy season in contrast to the dry season (*P* < 0.05) (Fig. 3).

### Enteropathogenic bacteria antibiotic resistance

Among enteric pathogens, a total of 93 colonies (64 % of positive isolates) were tested for antibiotic resistance. As shown in Table 4; 78, 50 and 35 % of the colonies were resistant to at least 1, 2 and 3 antibiotics, respectively. Most of the isolates were commonly resistant to ampicillin, nalidixic acid, trimethoprim–sulfamethoxazole and tetracycline. Although at lower levels, resistance to gentamicin and cefoxitin was also observed, particularly among *Salmonella* isolates (Table 5).

**Table 4:** Percentage of antibiotic resistance enteropathogenic bacteria (N = 93) and MAR index, at impacted La Paz River basin sampling points

Sample	≥1 (AR)^a^	≥2 (AR)^a^	≥3 (AR)^a^	MAR^b^ index
Water	76	45	36	0.21
Soil	79	50	29	0.20
Vegetable	85	69	46	0.27
All samples	78	50	37	0.21

^a^Percentage of enteropathogenic bacteria, resistant to a number of antibiotics

^b^Multiple antibiotic resistance index. Mean values of MAR Index were calculated for each type of sample (water, soil and vegetables) and across all samples

**Table 5:** Antibiotic resistance profile and MAR index of enteropathogenic bacteria isolated (N = 93), at La Paz River basin water, soil and vegetable sampling points

Enteropathogenic bacteria	Strain code^a^	Resistance profile	MAR index^b^
ETEC^c^	W2-1(LT/STh)	ST	0.17
	W2-2 (LT/STh)	C, TC	
	W2-3 (LT/STh)	AM, S, ST	
	W2-4 (LT/STh)	AM, NA	
	W2-6 (LT/STh)	NA	
	W2-7 (STh)	CI, NA	
	W2-14 (LT/STh)	AM, AB, S	
	W2-22 (STh)	NA	
	W2-51 (LT/STh)	AM, AB, C, TC	
	W2-61 (LT/STh)	–	
EAEC	W2-72	ST	0.18
	W2-75	AM, NA, S, ST, TC	
	W2-76	–	
EPEC	W2-38	AM, AB, C, S, ST, TC	0.33
	W2-60	NA, ST, TC	
	W2-74	AM, ST	
*Salmonella*	W2-9	NA	0.22
	W2-16	NA	
	W2-26	AM, C, CN, S, ST, TC	
	W2-35	AM, C, CN, S, ST, TC	
	W2-41	NA	
	W2-52	NA	
	W2-57	–	
	W2-90	AM, C, TC	
ETEC	W3-45 (LT/STh)	AM, AB, NA, S, ST	0.17
	W3-46 (LT/STh)	NA	
	W3-48 (LT/STh)	AM, C, TC	
	W3-73 (LT)	AM	
	W3-77 (LT/STh)	–	
	W3-78 (LT/STh)	NA	
	W3-79 (LT)	AM, ST	
EAEC	W3-8	AM, S, TC	0.24
	W3-20	AM, CN, S, ST, TC	
	W3-34	NA	
	W3-47	AM, ST	
	W3-80	AM, NA	
EPEC	W3-23	AM, ST	0.18
*Salmonella*	W3-10	NA	0.23
	W3-17	AM, C, CT, NA, S, ST, TC	
	W3-27	AM, AB, C, CN, FX, NA, S, ST, TC	
	W3-42	NA	
	W3-53	–	
	MV58	–	
	W3-65	TC	
	W3-91	TC	
ETEC	W4-15 (LT)	AM, C, ST, TC	0.21
	W4-33 (LT)	–	
	W4-49 (LT)	–	
	W4-50 (LT)	AM, CI, CN, NA, S, ST, TC	
	W4-62 (LT)	AM, C, ST	
	W4-89 (LT/STh)	NA	
	W4-88 (LT/STh)	NA	
EAEC	W4-21	–	0.23
	W4-25	–	
	W4-70	AM, AB, C, S, ST	
	W4-71	AM, AB, C, S, ST	
*Salmonella*	W4-12	–	0.20
	W4-18	AM, C, S, ST, TC	
	W4-30	AM, AB, C, CN, FX, S, ST, TC	
	W4-37	AM, CI, CN, FX, S, ST, TC	
	W4-44	–	
	W4-54	–	
	W4-59	–	
	W4-68	–	
	W4-93	–	
EIEC*/Shigella*	W4-69	AM	0.09
ETEC	S-5 (LT/STh)	AM, C, ST, TC	0.16
	S-13 (LT/STh)	NA	
	S-24 (LT)	–	
	S-31 (LT/STh)	NA	
	S-84 (LT/STh)	NA	
	S-85 (LT/STh)	AM, C, TC	
	S-86 (LT)	NA	
	S-87 (LT)	AM, ST, TC	
EPEC	S-19	AM, ST	0.18
*Salmonella*	S-29	AM, AB, C, CI, CN, NA, S, ST, TC	0.41
	S-43	–	
EIEC*/Shigella*	S-55	AM, FX	0.14
	S-56	AM, FX	
	S-66	–	
	S-67	AM, FX	
ETEC	C-32 (LT/STh)	AM, C, TC	0.23
	L-39 (STp)	AM, AB, C, TC	
	L-40 (LT/STh)	AM, C, TC	
	L-63 (STp)	AM, NA	
	L-64 (LT/STh)	AM, NA	
	L-81 (LT/STh)	AM, AB, C, TC	
	L-82 (STh)	NA	
	L-83 (LT/STh)	NA	
*Salmonella*	L-11	–	0.36
	L-28	AM, C, CI, CN, NA, S, ST, TC	
	C-36	AM, C, CI, CN, NA, S, ST, TC	
	L-92	–	

–, antibiotic sensitive strain

^a^Strain code was assigned based on the sample type and sampling site, followed by the numerical order of bacterial isolation: water (W), lettuce (L), chard (C) and soil (S) and sites (2, 3 and 4). No pathogens were detected at site 1

^b^MAR index calculated for each enteropathogenic category

^c^ETEC strains were characterized by presence of one of the toxin genes (LT/STh/STp)

MAR index values were comparable among water, soil and vegetable samples (Table 4), while in isolated pathogens varied from 0.1 in EIEC/*Shigella* to 0.4 in *Salmonella* (Table 5). Despite the fact that *Salmonella* strains presented the highest MAR values displaying resistance to as many as nine antibiotics, MAR index among pathogens did not show significant differences.

## Discussion

The impact of urban contamination, degrading the microbiological quality of La Paz River basin can be measured by the dramatic increase in the number of thermotolerant coliforms in surface water at all impacted sites. According to thermotolerant coliform data, site 1—not impacted by wastewater discharges-, can be classified as a source of water of maximum quality (Estado Plurinacional de Bolivia 1992), opposed to sites 2–4, that largely exceeded the standards for recreational water (i.e. 10,000 MPN/100 ml) (Osmond et al. 1997). Moreover, the average density at the agricultural area—site 3, surpassed in two orders of magnitude the allowed concentration for unrestricted irrigation water, according to WHO guidelines (WHO 1989) (i.e. <1000 MPN/100 ml). In this line, the density of thermotolerant coliforms found in fresh produce also exceeded the satisfactory microbiological hygiene criteria level 1000 MPN/100 g of fresh weight (ICMSF 1996). Overall, these parameters indicate that La Paz River basin at downstream locations aside from point 1, has a very poor microbiological water quality and is severely polluted by urban contaminants throughout the year. It is estimated that around 4.08 × 10^7^ m^3^/year of wastewater from municipal, industrial and hospital sources, is directly released into the river (Duran et al. 2003).

At impacted sites, the density of thermotolerant coliforms was associated with the presence of enteric pathogens. At least one pathogen was detected at every location in every water sample that contained levels of thermotolerant coliforms exceeding WHO water quality standards. This study demonstrates that collected water, soil and vegetables samples contained cultivable enteric pathogens potentially capable of causing gastrointestinal illness. At the region, farmer communities continuously use river water and sludge to increase food crop production. Therefore, vegetables may be contaminated with enteric pathogens while growing or upon harvesting. The fact that a high percentage of soil (10/12) and vegetables (8/12) samples were polluted with enteric pathogens, suggests that urban river pollution, predominantly from human sources contaminates agricultural fields. However, untreated manure and wild and domestic animals may also contribute to the pathogen load.

Most of the contaminated water and soil samples contained more than one pathogen, particularly ETEC and/or *Salmonella* which were present in all tested sites. At impacted water samples, the occurrence of multiple enteropathogens measured by the MEB index was higher during rainy season, particularly in the month of December, where at least four of the six tested pathogens were commonly present. Seasonal differences in enteropathogenic bacteria displaying an increased prevalence during the rainy season were also reported by Gonzales et al. (2013a, b) among diarrheal episodes.

Other studies (Salem et al. 2011) have also demonstrated the increased prevalence of *Salmonella* and ETEC in discharged wastewater compared to other DEC pathogens such as EAEC and EIEC. The marked prevalence of ETEC and *Salmonella* found in most of the impacted water samples may account for their higher survival rates in the environment. It has been shown that both pathogens can persist for several months in aquatic ecosystems by entering into a viable but non-culturable state (VBNC) (Lothigius et al. 2010; Santo Domingo et al. 2000; Waldner et al. 2012). Moreover soil and vegetables can act as reservoirs for *Salmonella* and ETEC (Islam et al. 2004; Singh et al. 2010), which attach to fresh produce by different mechanisms (Barak et al. 2007; Berger et al. 2009; Shaw et al. 2011). Both *Salmonella* and ETEC were identified as source of different foodborne outbreaks associated with the consumption of raw vegetables (Brendan et al. 2013; Yoder et al. 2006). Moreover ETEC is the leading cause of travelers’ diarrhea (Qadri et al. 2005). Overall, our data suggest that ETEC and *Salmonella* are the most resistant to survive in the environment and the ones that are likely mostly related to food and waterborne gastrointestinal infections in the region among the tested pathogens.

Therefore, the occurrence of multiple enteric bacterial pathogens (ETEC, *Salmonella*, EAEC and EPEC) at the impacted La Paz River basin area highlights the risk of microbial contamination, particularly with ETEC and *Salmonella* associated with irrigation water, agricultural soil, produce and vegetables consumption. Moreover, the continuous discharge of waste water run-off, generates concern that river may be one of the vehicles for transporting pathogens to downstream freshwater ecosystems of the Amazon macrobasin. Consequently, this may increase the risk of waterborne diseases among nearby riverine communities and agricultural settlements that rely predominantly on surface water as a primary source for drinking, fishing and swimming activities. Additionally, wild and domestic animals may be exposed to contaminated water upon drinking.

Interestingly, among diarrheal clinical isolates from acute diarrheal episodes in hospitalized children over a four year study (2007–2010) in Bolivia, EAEC was the most frequently detected followed by ETEC and EPEC (Gonzales et al. 2013a). These data support the fact that ETEC may have increased survival in the environment compared to other DEC pathogens. LT and STh were the most commonly found ETEC toxins among clinical strains (Gonzales et al. 2013b), contrary to what was found in this study, where LT/STh genotype were strikingly the most prevalent. Since it is commonly thought that the ETEC distribution in the environment is primarily related to human fecal contamination within the La Paz River, the potential exchange of plasmid carrying toxin genes among strains in the environment may not be excluded. Nevertheless, contamination of zoonotic origin cannot be excluded. Previous studies in Bangladesh reported a distribution of 67 % ST, 24 % LT–ST and 9 % ST among ETEC water isolates (Begum et al. 2007), similar to ETEC found in patients with diarrhea.

Among limitations of this study were the lack of data regarding the pathogen`s host source and the lack of quantitative information of the concentration (CFU/ml) of each tested enteric pathogen. Both data are relevant to better understand the level and origin of microbial pollution at the La Paz River basin area. In addition, pathogen occurrence most likely was underrepresented, since other important enteric pathogens such as *Vibrio cholerae*, *Campylobacter jejuni*, Diffuse-adhering *E. coli* (DAEC) were not tested.

The broad detection of antibiotic resistant enteric pathogens and the numbers of thermotolerant coliforms at La Paz river basin impacted area, raise concern regarding dissemination of multiple antibiotic resistant determinants into the environment. Antibiotic resistance genes are often inserted in integrons, transposons and plasmids, which facilitate their lateral transfer into a wide range of bacterial species (Nikaido 2009). Moreover, these data highlights the risk of acquiring upon exposure, antibiotic resistant bacteria directly from the environment, away from the health care settings.

Th**e** emergence and dissemination of antibiotic resistance, particularly multidrug resistance, among *E. coli* and other bacterial pathogens have become one of the most serious global public health threats (WHO 2015). In this study, enteric pathogens displayed multiresistance to antibiotics typically used in clinical settings. This may be due to the widespread use of several antimicrobial compounds in human therapy. In fact, among antibiotic resistance profiles, resistance to widely used antibiotics for the treatment of local diarrheal and acute respiratory infections (trimethoprim–sulfamethoxazole, nalidixic acid and ciprofloxacin) can be observed (Benguigui 2002). Moreover, resistance patterns are related to clinical DEC isolates found in previous years (Gonzales et al. 2013a; Rodas et al. 2005, 2011), suggesting that environmental and clinical pathogenic isolates may have a common origin. To address this issue, future studies should compare environmental and clinical isolates by molecular typing methods.

By comparing the antibiotic resistance among enteric pathogens with a previous report in the study area (Ohno et al. 1997) where most of the isolates were susceptible to ampicillin, nalidixic acid, tetracycline and chloramphenicol, it can be inferred that antibiotic resistance has increased within pathogenic bacteria isolated from the environment over time.

Overall, the high density of thermotolerant coliforms and presence of multiresistant enteric pathogens at impacted sites pose a risk for the emergence of new multiresistant pathogens.

## Conclusions

Within the La Paz River Basin area, river water is highly polluted by untreated wastewater run-off. High levels of thermotolerant coliforms and multiple enteropathogenic bacteria were detected along the sampling year at impacted water sites, irrigated soil and vegetables, with ETEC and *Salmonella* being the most prevalent. Moreover, 35 % of the total enteropathogens isolated were multiresistant to at least 3 antibiotics. These data highlight the microbial contamination of the La Paz River at the Amazon macrobasin emphasizing health risk of waterborne enteric pathogens transmission associated locally to the production and consumption of vegetables at the cities of La Paz and El Alto and in a larger extent to water intakes by the nearby Amazon riverine communities. Moreover, these results underline the need for improved guidelines and should prompt environmental governmental officials to develop effective prevention and control strategies to prevent and/or minimize the risk of foodborne and waterborne pathogen transmission in the region.

## Abbreviations

EHEC: *Enterohemorrhagic Escherichia coli*
EIEC/*Shigella*: *Enteroinvasive Escherichia coli* or *Shigella*
EAEC: *Enteroaggregative Escherichia coli*
EPEC: *Enteropathogenic**Escherichia coli*
ETEC: *Enterotoxigenic Escherichia coli*
MPN/100 ml: most probable number/100 ml
MPN/g: most probable number/g
stx1/stx2: Shiga toxin 1/Shiga toxin 2
ipaH: invasion plasmid antigen
paa: plasmid of adherence aggregative
eae/bfp: enterocyte effacement**/**bundle forming pili
lt/sth/stp: heat-labile toxin/heat-stable toxin human/heat-stable toxin porcine
invA: invasion gene A
MEB: multiple enteropathogenic bacteria index
MAR: multiple antibiotic resistance index
AM: ampicillin
AB: ampicillin–sulbactam
FX: cefoxitin
CT: cefotaxime
CI: ciprofloxacin
C: chloramphenicol
CN: gentamicin
NA: nalidixic acid
S: streptomycin
TC: tetracycline
ST: trimethoprim–sulfamethoxazole
